# Abstracts &c.

**Published:** 1889-11

**Authors:** 


### ABSTRACTS AND GLEANINGS.

---

**Chloralamid, a new Hypnotic.**—Chloralamid, manufactured on the suggestion of Dr. von Mering, of Strassburg, is the latest new hypnotic, and seems to possess many advantages over other similar preparations. It occurs in colorless crystals; is of a mild, slightly bitter taste, but is neutral; soluble in 9 parts water, and in 1½ parts alcohol. It is chemically affected by the presence of alkalies, but acidulated solutions can be administered.

Chloralamid is given in doses of 1 to 4 grammes, (the latter the maximum), in powder form or in solution with water or wine; the effect is asserted within half an hour and lasts from seven to nine hours. It has been successfully used by Drs. Hagen and Huefler, of Erlangen, in all varieties of sleeplessness, caused by nervous conditions, neurasthenia, phthisis, heart-failure, and in insomnia. It has also been employed and commendably noticed by the clinics of Strassburg, Greifswald, Giessen and Erlangen.—*Notes on New Remedies.*

**Death from Chloroform.**—Another death from chloroform is reported, this time from Cincinnati. The patient was a man suffering from an injured hand. At his own urgent request and against the advice of the doctor, he was given chloroform, a physician skilled in the use of anæsthetics administering the agent. Death occurred, as is so often true, after but a small quantity had been inhaled, and before he had sunk into anæsthesia. Every precaution seems to have been taken, and the case can only by recorded as one of those misfortunes to which any of us is liable in spite of the greatest care. Note that the operator, even in an apparently trivial case, had another and a competent physician with him. What a comfort, not to speak of the legal aspects of the matter, this must be to him now.—*Indiana Medical Journal.*

**Acetanilide in Epilepsy.**—Dr. Theodore Diller, of the State Hospital for the Insane at Danville, Pa. writes to the *Therapeutique Gazette*, June, 1889, that he has employed acetanilide in nine unselected cases of epilepsy. The remedy was administered three times a day, dry, upon the tongue; and the dose given was four grains, except in two cases in which this amount was doubled. It was given, with more or less interruption, during part of October, and all of December, January and February last. The month of October is not included in his estimate of the effect of acetanilide. Dr. Diller concludes that, in all cases in which the drug was given continuously, there was noted a reduction in the number of fits, ranging from twenty-five to seventy-five per cent, as compared with other months during which the patients were on the bromides and tonic treatments alternately. The remedy was in all cases well borne, producing no apparent mental or physical depression. This was in marked contrast with the depressant effects noted after a course of bromide treatment. No skin eruption was produced. In any given case, in which a great number of fits are occurring, and where it is desirable to control them as soon as possible, he thinks the bromides would be of far more value than acetanilide.—*Am. Prac. and News.*

**Treatment of Sciatica with Electricity.**—Dr. Max Weiss (*Cent. f. die ges. Therap.*) of Vienna, gives the following directions for the treatment of sciatica with electricity:

1. The annode (positive) is placed on the ischiatic region, the cathode over the different painful points; application stabile, for three to six minutes.
2. The annode placed on the different painful points, the cathode over the point of exit of the sciatic nerve.
3. One electrode is placed in the rectum, the other over the sacrum.
4. The application for many hours, even a day at a time, of a plate of copper, covered with flannel, over the sciatic region while a plate of zinc connected to the first by a metallic cord, rests on the posterior, superior or inferior surface of the thigh.
5. The different segments of the nerve (sciatic), from six to twelve inches in length, are treated successively from the sacrum to the foot, with the continuous current.
6. The faradic brush is applied throughout the whole extent of the posterior aspect of the member, after which stabile galvanization for ten minutes.—*Times and Reg.*

**Transplanting a Chicken's Cornea.**—The *Progres Medical*, July 13, 1889, announces—but rather skeptically—that Dr. Gravenigo, of the University of Padua, has been successful in an operation which has been tried hitherto in vain in France and elsewhere. This operation consists in grafting the cornea of a chicken upon a human eye. The graft was made recently, and the cornea is said to be very transparent, shining and convex.—*Med. and Surg Rep.*

**Dangerous Glycerine.**—Bismuth preparations sometimes contain small quantities of arsenic; in fact instances have been recorded where toxic symptoms of arsenic have been produced by the administration of bismuth. The recent investigations of Ritzert go to show that some of the samples of commercial glycerine contained large quantities of arsenic. Since these two preparations are very important therapeutic agents, it is well to remember these facts and get only the purest article.—*Ind. Med. Jour.*

**Sulphide of Calcium in Phthisis.**—Dr. Witherle (*La Clinique*) claims to have obtained good results in the treatment of phthisical patients by the internal administration of sulphide of calcium. He commences by giving a pill containing ¼ grain of the sulphide every two hours, and he gradually lessens the intervals between the doses until eructations or other symptoms of gastric irritation show that the limit has been reached. In most cases patients were able to take two pills every hour, and their general condition in every instance appeared to improve. This is, in reality, an indirect method of introducing sulphuretted hydrogen into the blood, and the principal is the same as that underlying Bergeon's treatment.—*London Med. Rec.*

**Treatment of Headaches.**—In acute headaches, with low vascular pressure, such as toxic headaches from alcohol and tobacco, exercise and food are potent remedies, and relief is obtained from cardiac stimulants, such as:

| | | |
|---|---|---|
| ℞ | Spir. ammon. aromat., | ½ dr. |
| | Spir. chloroformi, | 10 min. |
| | Aquam ad., | 1 oz. |

Antipyrin in 3 grain doses, repeated if necessary, is also useful. In chronic headaches of this character, where there is anæmia, iron is necessary; but as iron alone may cause headache, it is best to give 1 to 3 min. of tr. digitalis with it. If there is no anæmia, the digitalis alone will be found efficacious.

In acute high pressure headaches, the bowels must be kept open. Nitrite of amyl, carefully administered in very dilute state, is useful; but still more useful is nitro-glycerine. In anæmic girls, the last is particularly useful, given in doses of $\frac{1}{600}$ of a minim twice daily. Mix one minim of a one per cent. solution of the drug with water, in a six-ounce bottle, and give two tablespoonfuls as a dose. More than six doses should not be ordered.

In recurrent high-pressure headaches, alkalies are most beneficial, and when anæmia co-exists, iron is also indicated. It is best to give the iron with an alkaline carbonate as follows:

| | | |
|---|---|---|
| ℞ | Sodii bicarb., | 10 gr. |
| | Ferri et ammon. cit., | 5 gr. |
| | Spiriti chloroformi, | 10 min. |
| | Aquam menth. viridi ad., | 1 oz. |

M. Fiat haustus, ter die ante cibus semihora sumendus.

In persons of uric-acid diathesis a mixture of 20 grains citrate potash with 10 of salicylate of soda is useful.

Nitrate of amyl is useful in many cases of neuralgia of the scalp, where the patient's phraseology is, "when I comb my hair, I can't bear it.—*Practitioner.*

**Rules for Administering Chloroform.**—To be anesthetized, the patient should always be placed in a *horizontal* posture, preferably turned slightly to the right side. The clothing should be *loosened.* The stomach should be *empty.* The *fears* and *apprehensions* of the patient should be *allayed* as much as possible. The chloroform should be *pure.*

The administration should *begin slow* and *gradual* manner, and an abundant supply of fresh air being mixed with the chloroform vapor during all the anesthetic procedure; and the patient instructed to breathe in a slow, easy, natural manner.

Never fully anesthetize a surgical patient while he is in an excited condition.

Never operate, however short or trivial the operation, under *partial* anesthesia.

Do not examine the *heart immediately before*, or feel the *pulse during* the early stages of anesthesia; but watch carefully the force, frequency and quality of the respiration.

Never maintain anesthesia longer than is absolutely necessary but *repeated* short anesthesias; *e. g.* as for extracting several teeth at short intervals, are more dangerous than one continuous properly maintained anesthesia of reasonable duration.

The movements of hiccough and swallowing should be watched for, and if they occur, they evidence that the anesthetic is being given too rapidly.—*Nashville. Jour. Surg.*

**Antiseptic Power of Salol.**—At a recent meeting of the Hunterian Society, Mr. Corner introduced a series of cases illustrative of the antiseptic power of salol (salicylate of phenol)as a dressing for wounds, after the part had been rendered aseptic by a 1 in 20 solution of carbolic acid. He did not claim for it greater power than iodoform, and probably other antiseptics, but it has advantages over some. It possesses a pleasant aromatic odor, can be used freely without fear of irritation or poisoning, is absorbent of moisture, which drying forms a hard but friable covering. It will prevent putrefaction; it will not destroy it when once established. It has been used in increasing frequency for several years at the Poplar Hospital, and with excellent results, in compound fractures and dislocations, also as a dressing in amputations, minor and major, and in compound comminuted and depressed fractures of the skull. The first case shown was a compound comminuted depressed fracture of the frontal bone, in which the bone was elevated and some spicules removed. Afterwards the wound was washed with a solution of carbolic acid (1 in 20), the opening filled with salol, and a drainage-tube inserted.

The dressing was undisturbed for fourteen days, remained sweet, and healed on the twenty-sixth day. His temperature remained from the first under 100°. A second case treated in January, 1880, was a compound fracture of the olecranon, head of radius, and humerus, opening the elbow joint, with considerable damage to soft parts, the elbow having been crushed by the passage of a railway engine over it. The olecranon was splintered and drawn up, causing serious tension of skin and necessitating removal of both portions. The antiseptic treatment and dressing were the same as in the previous case, but required changing after four hours and again next day, in consequence of oozing through. The parts were then left untouched for thirty days. The temperature went up the day after the injury, and remained about 101° for three days, 100° for two days, and then fell to normal. Two other cases were shown: one a crushed compound fractured finger, dressed twenty-one days before, and not exposed since, there having been neither pain nor elevation of temperature; the other was a compound fracture of first phalanx of finger, only dressed at the time of the accident, and left undisturbed for a month, when it was found perfectly healed. It was pointed out that this was the common experience in such cases, and that even if gangrene followed the parts remained sweet.—*Lancet.*

**Infantile Diarrhœa.**—The medical treatment is divided to meet the demands of three sets of cases.

1st. Those with vomiting, colic, convulsions, frequent greenish stools and great exhaustion. At the onset give a tablespoonful of the following mixture:

| | | |
|---|---|---|
| ℞ | Ol. ricini, | 1 oz. |
| | Glycerin, | 2 dr. |
| | Ol. cassiæ, | 1 drop. |

After it has operated freely, give some antiseptic combined with a small dose of opium. Salicylate of sodium is perhaps the best. For a child two years old the correct formula will be:

| | | |
|---|---|---|
| ℞ | Sodii salicylatis, | 4 grains. |
| | Tr. opii deod, | 10 drops. |
| | Syr. simp., | 1 oz. |

M. Sig. One teaspoonful every three hours.

The result is rapid and satisfactory.

2d. The diarrhœa may be frequent and of a vivid grass green color but unattended by vomiting and marked prostration. This variety is rapidly cured by lactic acid. A 2 per cent solution may be given in doses of one teaspoonful every one or two hours.

3d. This type is commonly insidious in origin; the stools being softer and more frequent than usual for a long time before the onset of alarming symptoms; or it is engrafted on a pre-existing cholera infantum. Fully developed, the stools are green or pale in color, moderately thin, and containing sago like pellets of mucous with here and there specks and streaks of blood. Later on we find shreds and strings of mucous like substance apparently caused by sloughing from superficial ulcers of the colon. Examine carefully into the sanitary surroundings of the patient and eliminate all errors in diet. A moderate dose of castor oil will remove all irritating matter from the intestines. Then a small dose of opium and bismuth subnitrate will quiet the nervous system and soothe the intestinal mucous membrane. As soon as the number of stools are reduced to a moderate number, small doses of Fowler's solution of arsenic may be added, and the opium gradually discontinued. Bloody stools are frequently corrected by injections of nitrate of silver; one grain to the pint; at intervals of twelve to twenty hours. When convalescence is established a sojourn to the seaside is advisable.—*N. E. Med. Monthly.*

**Is Antifebrin Dangerous?**—Dr. A. L. Saylor, in answer to the above question, writes as follows: "Various authorities agree in pronouncing antifebrin a safe and reliable antipyretic, useful in quickly reducing temperature without any untoward results. Yet my experience does not justify me in coinciding with my contemporaries, as results will show. Having had personally but limited experience with the drug, I concluded, recently, to test its properties in a case of infantile fever by administering a 4 grain dose to a child of three years of age, whose temperature persistently remained at from 101° to 103°. Hardly had one-half hour elapsed when I was summoned to the bedside to find that the dose had done its work effectually, as the temperature was below normal, with impending collapse indicated by cold extremities, feeble respiration, and labored heart's action. Prompt administration of restoratives prevented the impending result, and the patient was eventually restored to health, but under other treatment than that afforded by antifebrin. As no organic heart derangements existed, and the drug was supposed to be a prime article, we are at a loss to account for its peculiar action in this instance, unless some idiosyncrasy of the patient existed, or the much vaunted antipyretic properties of the drug are of a dangerous character. If so, it should be administered with circumspection."—*Times and Register.*

[The dose here was simply too large for the child—should have been given in 1 gr. doses every hour, until its effects were observed. —ED. RECORD.]

**Cascara Sagrada and Glycerin in Constipation.**—Dr. Geo. E. Turrill, of Wausen, Ohio, writes: The superiority of cascara cordial over the fluid extracts in regard to bitter principles is great, and I have used it almost constantly during the past eight years with excellent results. I believe we possess nothing in medicine better adapted to relieve constipation in general, and especially that peculiar to pregnancy. There are cases, however, in which it has been a failure, neither the time of taking or size of dose making any material change. After noting these facts it occurred to me that the addition of glycerin might prove a valuable auxiliary, accordingly I prepared a mixture, consisting of four-fifths of the cordial to one-fifth of glycerinum, administering as before. The results have been excellent. I frequently see cases of extreme irritability of the mucous membrane of the bowels, and in these conditions I believe the glycerin quiets the irritability and accelerates the action of cascara. Permit me to cite a few cases:

Mrs. A. Cancer of rectum and severe constipation with great pain; cascara cordial given as above; results remarkable. Not only was the constipation relieved, but the pain lessened and gradually the ulcerating surfaces healed.

Case 2. Mrs. Y. Rectal ulcer with hemorrhoids; constipation extreme. Prescribed cascara and glycerin. The constipation is removed, the hemorrhoids improving, and the ulcer healing.

Case 3. Miss J. Diabetes. Nothing relieved the constipation until glycerin and cascara cordial were employed.

I might mention others, but these will suffice for the present.—*Med. Age.*

**Mullein and Vinegar in Phlegmasia Dolens.**—Dr. Ludlum (*Times and Register*) reports the successful treatment of phlegmasia dolens as follows:

Remembering that when a boy on the farm the older brothers used mullein (verbascum thapsus), boiled in cider vinegar, on the horses to reduce swollen parts, that I conceived must be in some degree similar to this (and my prejudice being somewhat disarmed since it was recommended for consumption), I ordered a trial of it on this patient, and the result was most gratifying, as in a few days' use by fomentations she was comparatively comfortable, and the limb reduced nearly to the size of the other, and the tissues nearly as soft. She made a good recovery.

**Rheumatic Fever.**—Longstreth presented a young man, aged twenty-three, salesman by occupation, suffering from rheumatic fever. There was a history of rheumatism in his family, his father being a sufferer from it, and his mother having heart disease. Five years ago he had a slight attack, and a year later a much severer one. The present attack had been induced by exposure. When admitted to hospital his joints were all swollen, and a high fever prevailed. Ausculation disclosed a double cardiac murmur, aortic and mitral. Lungs were negative, bowels constipated. He was given ten-grain doses of salicylic acid every hour, but without effect—neither the temperature nor the swelling in the joints being reduced. He was then placed on acetate of potassa, ten grains, and bicarbonate of soda, twenty grains, every two hours, which also proved to be of no avail. Finally, the iodide and bromide of potassa were given, ten grains of each for a dose every four hours, from which very satisfactory results were obtained. The joints were smeared with blue ointment.

This patient, a woman, who works out when she is able, presented upon admission some evidences of typhoid fever. The wedge-shaped area of redness at the apex of her tongue was distinctly marked. Her temperature was 104.8°. The abdomen was resonent and irritable, and patient complained of having much diarrhœa. The evacuations were chunky, with hard, fecal masses, but were not of the characteristic yellow color. Patient also vomited, which ceased upon giving bismuth. Micturition was difficult. I ordered her bismuth in ten gr. doses every two hours, also quinine to reduce temperature, and a milk diet. A cold flaxseed poultice was applied to the abdomen. Hot poultices increase the temperature 1° to 2°, which was to be avoided in this patient. I continued the quinine, in three-grain doses, night and morning, and gave enough bismuth to keep the stool black, so as to allay the irritation of the mucous membrane of the intestinal tract —*Longstreth.*—*Ib.*

**Beetroot in Habitual Constipation and Hemorrhoids.**—In the St. Petersburg new periodical *Meditzina*, No. 6, 1889, p. 10, Dr. S. Kazatchkoff draws attention to the fact that a strong infusion or decoction of the common beetroot (*Bela vulgaris;* Russ, *buraki* or *sviokla*) represents an excellent mild aperient, very much in favor with the South Russian peasantry, who resort to it especially in cases of atonic habitual constipations and hemorrhoids. It is taken in doses of from half to one tumblerful at bedtime or early in the morning about an hour before breakfast. The remedy does not cause any abdominal pain, griping or rumbling, nor does it create any tendency to consecutive constipation. On the contrary, any disposition in that direction is decidedly removed by a daily use of the decoction for a certain period. It is stated, however, that the patient's bowels get habituated to the beetroot in a week, so that, by the end of that time, the dose of the decoction should be increased, or a couple of apples a day be added. According to the author's experience, many constipated patients prefer the beetroot "juice" to castor-oil, rhubarb, podophyllin, magnesia, milk-sugar, milk, mineral waters, and similar ordinary means, used by them previously to their making acquaintance with the simple remedy under consideration.—*London Med. Recorder.*

**Rheumatism.**—An excellent remedy for either articular or muscular rheumatism or acidism. The following remedy for rheumatism or acidism, having given great satisfaction for many years, is forwarded you for publication :

| | | |
|---|---|---|
| ℞ | Lithia citrate | 1 ounce. |
| | Potass. citrate | ½ ounce. |
| | Water | 8 ounces. |

M. Sig. Take a teaspoonful four times a day or every two hours. Please give it a trial and report.—Dr. Luigi Galvani Dvane, in *Med. Brief.*

**Hydrate of Chloral in Chapped Nipple.**—(Trans. in the St. Louis Med. and Surg. Jour.)—Dr. Ivan A Mitropolsky, Moscow, recommends chloral as an excellent local means for fissured and excoriated nipples. The latter should be kept covered with compresses

(soft linen) soaked in a solution of half a drachm of chloral in three ounces of water. The compresses should be changed every two and a half or three hours. When a prolonged application is necessary, it is advisable to use a weaker lotion (one-half drachm to six ounce). The solution leaves a thin, whitish, firmly adherent film over the diseased surface, which does not disappear by suckling. Pain and tenderness are said to be strikingly relieved almost immediately: the lesions rapidly healing. The chloral compresses do not produce any bad effects on nurslings.—*Arch. Gyne.*

**Scarlet Fever—Diphtheria.**—Dr. Herr gives an account of an epidemic of scarlet fever and diphtheria that was treated with no other remedy than yeast, the scarlet fever running a mild course, and the membrane in diphtheria being cast off with remarkable quickness, while heart-failure and paralysis in no case made an appearance. A medium dose was a dessertspoonful hourly, assisted with washing out the mouth and fauces with a wash consisting of one teaspoonful of yeast to five of water, at intervals of every two hours.—*Ex.*

RETROSPECTIVE THERAPEUTICS, BY ALFRED K. HILLS, IN MED. TIMES.

**Nutmegs.**—Dr. J. V. Shoemaker (*Med. Bull.*) says that the medicinal qualities of nutmegs are worthy of more attention than they have hitherto received. They will be found valuable in the treatment of summer diarrhœas, many cases quickly yielding to the administration of half a drachm in milk. Insomnia may be effectually relieved by them when opium has failed, and chloral is contra-indicated. They can be administered in delirium tremens with safety and benefit when any other sedative would be perilous. An ointment composed of two drachms of powdered nutmegs, one drachm of tannic acid and one ounce of lard, constitutes an excellent application for itching and irritable hemorrhoids. The dose of powdered nutmeg varies from ten grains to two drachms for adults. Larger quantities than this have produced profound coma lasting for hours.

**Hamamelis in Hemorrhoids.**—Rohe treats internal hemorrhoids with fluid extract of hamamelis and glycerine; half a drachm of each every four hours. The remedy is promptly effectual in uncomplicated cases.

**Cascara Sagrada in Rheumatism.**—Dr. H. T. Goodwin, of the U. S. Marine Hospital service, claims to have accidentally discovered in cascara sagrada a specific against rheumatism. Exhibiting the drug first upon himself, he afterwards gave it in thirty cases of rheumatism of all types and grades, with curative effect in every case. All other remedies were discontinued. He usually combines it with syrup in equal parts, and instructs the patient to take from thirty to forty drops in water.

**Ferrum Phos. in Pneumonia.**—Professor Goodno reports three cases of croupous pneumonia aborted with ferr. phos., after developing the crepitant rale and rust colored sputa. The disease went no further, but recovered by lysis, not by crisis as usual.

**Glycerine Suppositories for Constipation.**—We have already directed attention to the injection of small quantities of glycerine into the rectum as a means of overcoming constipation. This method, somewhat extensively employed in Germany, is often by no means convenient to carry out, because few syringes are available for injecting such small doses as fifteen drops of any medicament. A better idea has been suggested by Boas, in the *Deutsche Med. Wochenscrift*, namely, that the glycerine be given in suppositories or capsules, and Messrs. Parke, Davis & Co. furnish the suppository.

This method (as the *Med. and Surg. Reporter* remarks) is simple, easy of execution, and, if it is what it is claimed to be, must be very superior to the use of enemata, or of purgatives given by the mouth.

**Stannum in Headache.**—Dr. Cyrus M. Babcock, in the *American Homœopathist* for October, relates an interesting case of headache cured in three days by the use of stannum 3x trituration. The patient was attacked four or five times a year with pain over the right eye, which would last for several days. The pain was so intense as to prevent work. The pain was intermittent and had something of the stannum cecendo et diminuendo style about it. Sulphate of morphia gave very little relief. Dr. Babcock has cured a number of chronic headaches with stannum, and thinks the remedy is too infrequently prescribed.

**Antiseptic Treatment of Diphtheria.**—After a trial and study of the many remedies recommended in diphtheria, Dr. LeGendre, chief of clinic, Hospital of Infants (*Archiv. Laryng.*, October, 1887), advocates the following treatment:

Touch three or four times a day the whole extent of the membrane and a short distance below it with a one per cent solution of the bichloride of mercury in alcohol.

Every two hours spray freely with a warm saturated solution of boric acid (four per cent). Internally, give sodium benzoate in quantities of from forty-five grains to three drachms per day, with claret or champagne and coffee. This treatment has given excellent results at the Hospital of Infant's Diseases.

The author insists that caustic applications to the throat are never necessary, and that remedies which might derange the digestive functions should never be given.

**Sea-Water Spray.**—Prof. P. Mantegazza considers the local application of a spray of sea-water to the fauces and larynx as particularly beneficial in chronic non-specific laryngitis, in obstinate catarrhal pharyngitis, in bronchial catarrh, in tuberculosis of the first and second stage, and in various forms of scrofula. The first effect of the spray is a gentle excitement, followed by a greatly increased activity and appetite. This indirectly promotes improvement. In local affections of the larynx, etc., the spray seems to exercise a directly sedative and specific effect.

**Strychnine in Alcoholism.**—Korona, of Tiflis, summarizes his results as follows:

1. In acute intoxication strychnine is completely inactive.
2. In ten out of eleven alcoholic patients, after three or four injections of the drug, there appeared an aversion to brandy.
3. It is the most effective remedy for dipsomania, but the treatment must be repeated in course of time.
4. It is also a very good remedy for chronic alcoholism, when it is accompanied with such symptoms as neuralgic pains in the lumbar region and calf of leg, tabetic gait, trembling of hands, etc. A hypnotic effect of the drug is also very pronounced.
5. The remedial value of strychnine is very slight in cases of alcoholism unaccompanied by any symptoms.
6. Strychnine does not possess any accumulative action, so far as alcoholists are concerned.

**Gossypium Herbaceum.**—A tincture made from the fresh inner bark of the root of the cotton plant, and subsequent potentizing, gives us a remedy containing a principle similar in its action to that of secale cornatum (*Pop. Zeit. fnr Pom.*). Gossypium will often be indicated and prove successful in the morning vomiting of the pregnant; in uterine hemorrhage and painful menstruation; and in female sterility from too scanty menstruation.

**Cocoanut as a Tænifuge.**—Professor Parisi, of Athens, has discovered, by numerous experiments on himself and others, that the ordinary cocoanut is a very effectual remedy against tape-worm. He orders the milk and the pulp of one cocoanut to be taken early in the morning fasting, no purgative or confinement to the house being required, and suggests that pharmacists should make cocoanut preparations which might answer the same purpose, and, perhaps, prove rather more convenient.

**Modelling Clay in Mastitis.**—Dr. E. L. Maizel mentions in the *Vrach*, No. 21, that he has for some time employed modelling clay as an application in mastitis, with excellent results. The idea was obtained from Dr. S. Lukashevich, who employed white modelling clay in epididymitis. Dr. Maizel has made use of it in twelve cases, seven of parenchymatous inflammation, of which only three suppurated; and five of phlegmonous inflammation, of which only one went on to form an abscess. The pain was rapidly alleviated and the fever diminished by this treatment, which, if commenced sufficiently early, appeared to cause the inflammatory process to terminate in resolution without the formation of pus. When abscesses had formed and broken, the wound rapidly healed, and the indurated lobules returned to their normal condition without suppurating, the clay proving a much less troublesome treatment than Kiwisch plan of starched bandages, and much less painful than the application of collodion, as recommended by Latour and Sprengel, being at the same time quite as satisfactory in its results. This method is especially suitable for country practice, as white modelling clay is a very common and cheap substance, and patients take to the treatment very kindly, the application being always grateful to them. The capacity for heat possessed by clay being considerable, it answers the purpose of ice-bags or cold compresses, and is greatly preferred to them by patients. The method of application is simple enough. The breast is first washed with a sponge and a piece of soft gauze applied to it. Another piece of gauze is then cut to the proper size, an aperture being made for the nipple, and an even layer of well-mixed clay, free from lumps, spread over it. This is then applied to the breast over the first piece of gauze, and secured by a bandage passing over the shoulder of the opposite side. The dressing is renewed night and morning. This kind of treatment is also applicable in distressing fullness of the breasts, when for any reason the patient cannot suckle the child. The secretion is arrested, and the fullnes and tenderness often disappear in the course of twenty-four hours.

**Bromidia as a Hypnotic.**—The success which this drug has achieved in France is somewhat remarkable. The French as a nation are remarkably conservative in everything save their politics, adhering tenaciously to the ideas and objects with which they are familiar, and regarding with corresponding suspicion all novelties and inovations, especially those coming from abroad. Hence it is that the materia medica of France has not marched *pari passu* with that of its neighbors. The bromidia (Battle) at once attracted the attention of the French physicians, and their experience with it so developed their confidence in it as a prompt, reliable and harmless hypnotic that, in utter disregard of all that they had been taught and believed respecting the danger and unreliability of alien products, they promptly accorded it a place in their repertoire of remedial agents, and are now using it as freely as any medicinal preparation included in the Codex. In no other country, in fact, does it enjoy a larger measure of popularity than in France, and so great is the demand for it that it has been found necessary to manufacture it here in large quantities in an establishment especially arranged and organized for that purpose.

As no extraneous influences have been brought to bear in its favor it has had to make its own way in the face of opposition and prejudice of the most formidable character, upon the strength alone of its virtues as a remedy for insomnia and other corresponding disturbances of the nervous system, the conclusion is legitimate that it really possesses the therapeutical properties claimed for it, that it is a hypnotic *par excellence*, and without a rival.

To those familiar with the use of bromidia (Battle) no argument like this is necessary, for it speaks for itself by fulfilling the indications for which it is administered with a certainty, efficiency and harmlessness which elicit at once the delight of the prescriber, and give to the profession the assurance of possessing one remedy at least which approximates so near to infallibility of action as to justify the title of *specific.*—Edward Warren-Bey, M. D., C. M., LL. D., in *Press and Circular*, London, Eng., March 27, 1889.

**Nitrite of Amyl in Epilepsy and Purtussis.**—Some years since, the late Dr. David W. Yandell, of Louisville, Ky., suggested the employment of amyl nitrite as an anti-spasmodic, and incidentally remarked its internal administration might prove of value in some forms of epilepsy, especially if it could be given in a full dose at the first evidence of an *aura*. So far as known, however, this suggestion has met with little attention, and the drug administered only in cases of *status epilepticus*, though a moment's consideration of its physiological action could but lead to the conclusion it might be satisfactorily employed in simple convulsions. Further affirmative evidence was had by the writer through actual administration by inhalation to a convulsed (epileptic) canine, but opportunity has not since offered for experiment in the human animal. Recently, however, Dr. J, P. Parkinson, of London (Eng.), writes the British Medical Journal that, being called to a very severe fit in a young man who had been epileptic from birth, and whose mind had been so impaired that, though twenty-one years old, he was unable to read, write, or dress himself, he determined to try amyl nitrite.

"After inhalation of one capsule, the convulsion ceased, and in about twenty minutes the patient was himself again, the insensibility which follows such fits being almost nullified.

"Since then the drug has been tried four times, and each time with success; if administered at the commencement of the fit, the latter was aborted, and the insensibility almost completely prevented. This patient is in the habit of having, on an average, a fit daily, and frequently a succession of fits—status epilepticus. He has been a patient for years at the Epileptic Hospital, Queen's Square, and has been taking bromides daily, but apparently with no effect."

Though the action of amyl nitrite has not been worked out in this instance, it is more than probable, like glonoin (trinitrin), it is due to its dilating influence upon cerebral arteries contracted during convulsions.

The marked anti-spasmodic influence exerted by the drug, led the writer some years since to employ in pertussis, and with most happy results, as it rarely, if ever, failed to cut short the cough paroxysm if administered promptly at the first indication of an onset; a teaspoonful of the following being employed for a child of six years:

| | |
|---|---|
| Amyl nitrite, | 0.8 grammes. |
| Spts. vini recti, | 5.8 grammes. |
| Glycerini | 9.7 grammes. M. |

This formula has a wide range of applicability, and is of value in spasmodic asthma, torticollis, pseudo-membranous laryngitis, etc. In fact, the benefits to be derived from internal administration of amyl are scarcely appreciated, and the drug has never received the attention it deserves.

**The Congress of American Physicians and Surgeons.**—Dr. C. H. Mastin, of Mobile, who may be said to be the father of this organization, set a wholesome example in the ground he took, at a recent meeting of the Executive Committee, for declining the nomination to the presidency. He is reported as having said that no member of the Executive Committee ought ever to be elected to the presidency. Thereupon Dr. S. Weir Mitchell, of Philadelphia, was chosen president, and Dr. William H. Carmalt, of New Haven, secretary. The next meeting is to be held in Washington, in September, 1891.—*Ib.*

**Tannin Treatment of Phthisis.**—Dr. E. Houze, of the Hospital St. Jean, Brussels, after having tried the tannin treatment on all his phthisical patients for the last year and eight months, states as the result of his observations that it gives excellent results in all stages of the disease, and especially in the condition where cavities exist. Indeed, he has no hesitation in declaring that of all the different kinds of treatment for phthisis which he has tried this has given by far the most encouraging results. The dose he employs ordinarily is fifteen grains, which quantity is taken three times a day. It is, as a rule, well borne; where this is not so, it is ordered to be taken with meals. After the first few days the expectoration and the sweats diminish, the cough decreases, and in many cases the appetite undergoes a marked improvement. The majority of the patients suffered from some slight degree of constipation, though in some this feature was sufficiently marked to require treatment; while others, again, suffered from diarrhœa. The character of the expectoration changed for the better, the sputa becoming white and frothy instead of green and firm. In some cases the diminution of the expectoration was followed by increased dryness of the cough, so that the patients complained that it fatigued them more; this was easily remedied by prescribing a few spoonfuls of syrup of codeia. The physical signs underwent a remarkable change for the better, at least those depending on auscultation, moist rales giving place to dry rhonci, and large gurgling rales decreasing progressively until they gave place to mere blowing respiration. These changes were evidently due to the drying up of the cavities, in consequence of which the hectic present in many of the cases vanished, the patients increasing considerably in weight and gaining strength in a remarkable manner. The percussion signs were not found to undergo so marked a change as those depending on auscultation, but even here some improvement could be detected. No bacteriological observations were made.—*Lancet.*

